# Smart nanocarriers for cancer: harnessing exosomes and lipid systems in photodynamic and immunotherapy

**DOI:** 10.3389/fimmu.2025.1687953

**Published:** 2025-10-17

**Authors:** Amrita Swain, Soumya Ranjan Jena, Luna Samanta

**Affiliations:** ^1^ Redox Biology & Proteomics Laboratory, Department of Zoology, Ravenshaw University, Cuttack, India; ^2^ Center of Excellence in Environment & Public Health, Ravenshaw University, Cuttack, India

**Keywords:** exosomes, nanocarriers, photodynamic therapy, immunotherapy and cancer, liposomes

## Abstract

Cancer remains the leading cause of death worldwide. Despite decades of continuous research, limitations persist in existing therapeutic approaches. Conventional strategies such as surgery, chemotherapy, and radiotherapy, though advanced, face challenges including poor bioavailability, toxic side effects, inadequate targeting of cancer cells, and limited survival benefits. The major issue lies in the inability of improved drug formulations to effectively reach cancer cells. Emerging approaches such as photodynamic therapy (PDT) and immunotherapy have shown greater promise, offering reduced side effects and higher treatment efficiency compared to traditional methods. Various natural and synthetic nanocarriers, including exosomes, liposomes, solid lipid nanoparticles (SLNs) and micelles have been explored as drug delivery vehicles in these therapies. Among them, exosomes, being natural secretory vesicles, have shown unique potential as independent delivery systems. However, challenges and limitations remain in their application for precise cancer targeting. A combinational strategy, integrating exosomes with other lipid-based drug delivery systems (LBDDS), while preserving their intrinsic properties and engineering their surface to carry photosensitizers (PS) or immune modulators, could overcome these barriers. Such well-designed natural cargos may enhance therapeutic efficacy, modulate the tumor microenvironment, and address current shortcomings in cancer therapy. This review highlights the individual applications of PDT and immunotherapy using exosomes and LBDDS, and explores their potential synergistic use for more effective and targeted cancer treatment.

## 1 Introduction

Cancer remains the leading cause of death worldwide, significantly impacting human health and well-being. While traditional treatment strategies, such as surgery, chemotherapy, and radiotherapy, have advanced over the years, they often come with severe side effects, poor tumour targeting, and limited survival rates (1). These limitations have driven the exploration of more modern treatment approaches, including photodynamic therapy (PDT) and immunotherapy, both of which show considerable promise in enhancing treatment efficacy while minimizing adverse effects.

Photodynamic therapy (PDT) has made notable progress, particularly in treating solid tumours. Photodynamic therapy (PDT) involves the administration of a photosensitizer followed by localized irradiation with light of a specific wavelength, generating reactive oxygen species (ROS) that selectively induce cytotoxicity in targeted cells (2–5). These ROS cause apoptosis, DNA damage, and immune responses at the tumour site. However, the clinical application of PDT faces several obstacles, such as poor solubility, aggregation, and off-target effects of PSs. Therefore, developing efficient and safe drug delivery platforms is crucial (6). Despite its potential, PDT is still challenged by off-target effects, necessitating the development of optimized delivery systems that ensure precise tumour targeting while minimizing unwanted impacts.

Immunotherapy, which harnesses the body’s immune system, often in combination with monoclonal antibodies, has emerged as a promising cancer treatment. By stimulating the immune response, immunotherapy aims to target and eliminate cancer cells. However, its therapeutic potential is limited by challenges such as off-target delivery, immune tolerance induction, and immune evasion by tumours (7, 8). Additionally, the hypoxic and immunosuppressive characteristics of the tumour microenvironment (TME) further reduce the efficacy of these therapies (8). The TME’s ability to induce immune tolerance and evade immune surveillance poses significant barriers to effective immunotherapy, making it imperative to overcome these obstacles to improve cancer treatment outcomes.

Exosomes, naturally occurring extracellular vesicles (EVs) with liposome-like bilayer structures, have shown great promise in cancer therapy due to their prolonged circulation time, immune system evasion, and tumour-homing capabilities. These properties make exosomes ideal candidates for targeted drug delivery (6). Moreover, exosome-based cancer immunotherapy has emerged as a promising strategy to combat the immunosuppressive TME, engage immune checkpoint blockades, and deliver cancer vaccines (9).

In recent years, biomimetic drug delivery systems (BDDSs), such as lipid-based nanocarriers (liposomes, nano-emulsions, solid lipid nanoparticles, nanostructured lipid carriers, and lipid-polymer hybrid nanoparticles), have attracted significant attention for their ability to deliver therapeutic agents including PSs with greater precision and efficiency. Lipid-based systems are particularly appealing due to their enhanced biocompatibility, solubility, and permeability (10, 11). These systems improve the bioavailability of hydrophobic and lipophilic drugs, making them versatile in delivering both hydrophobic and hydrophilic compounds (12, 13). Despite these advancements, precision targeting of tumour cells while minimizing systemic side effects remains an active area of research. Although preclinical studies have demonstrated the promise of exosome and lipid-based systems, their clinical translation is limited by challenges related to production, scalability, and stability. Addressing these challenges is crucial for unlocking the full potential of these systems in clinical cancer treatment.

This review explores the application of exosomes and lipid-based systems for the delivery of therapeutic agents in PDT and immunotherapy. It examines the potential advantages of these innovative drug delivery platforms, identifies current limitations, and outlines promising future directions to overcome these challenges in cancer treatment.

## 2 Exosomes as drug delivery vehicles

**Figure 1:**
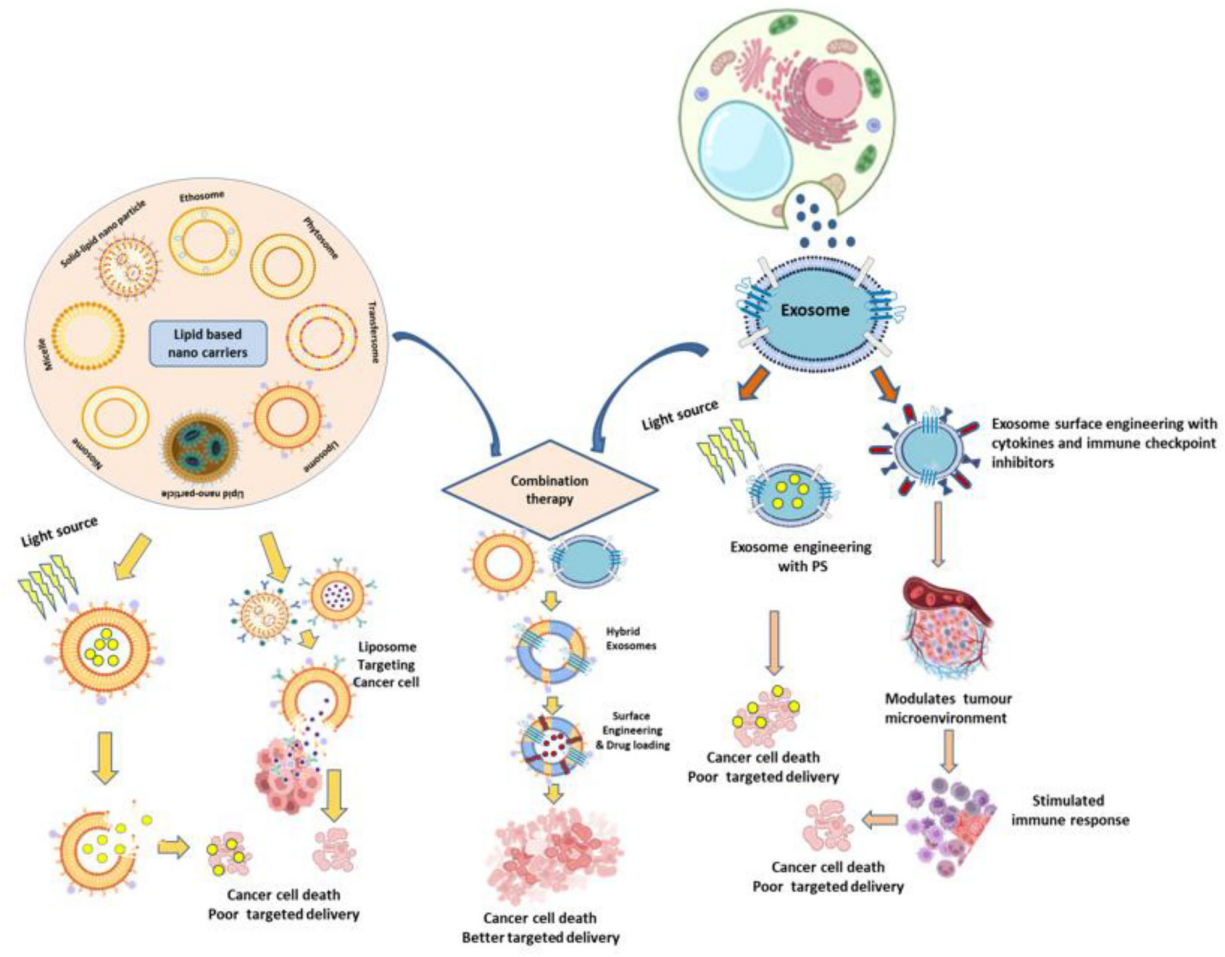
Schematic representation of combined exosome and lipid-based nanocarrier strategies for cancer therapy, integrating photodynamic therapy (PDT) and immunotherapy to achieve improved therapeutic efficacy compared to conventional treatments.

Exosomes are a specific type of nanosized extracellular lipid bilayer membrane vesicles secreted by almost all cell types and play a pivotal role in intercellular communication (**Figure 1**) (9, 14). In 1990s from immunological studies by Raposo et al. (1996) the role of exosomes in adaptive immunity was established by demonstrating secretion of exosomes B lymphocytes which are capable of antigen presentation to T cells, carrying functional MHC class II molecules (15). Shortly thereafter, another report by Zitvogel et al. (1998) exhibited that dendritic cell-derived exosomes could prime cytotoxic T lymphocytes and eradicate established murine tumors, thereby introducing exosomes as a novel platform for cancer immunotherapy (16). Building on these discoveries of exosomes, their therapeutic potential as a drug delivery vehicle was later established by Alvarez-Erviti et al. (2011), who commenced targeted exosome engineering to deliver siRNA systemically across the blood–brain barrier. All together, these pioneering studies laid the foundation for the broad exploration of exosomes in immunotherapy, oncology, and nanomedicine (17).

### 2.1 Biogenesis and characteristics

Exosomes, typically ranging in size from ~30 to 150 nm, are characterized by their ability to encapsulate various biological molecules, including nucleic acids and proteins, within their lumen or lipid bilayer. These vesicles are released from cells under both physiological and pathological conditions to facilitate cell-to-cell communication and enable cargo transport both *in vivo* and *in vitro*. The biogenesis of exosomes occurs either constitutively or can be induced by external stimuli, originating from endosomal compartments. Endosomes give rise to three primary types of vesicular structures: macrovesicles (50–1000 nm in diameter), which are shed from the cell membrane through membrane remodelling and outward blebbing; apoptotic blebs (100–5000 nm), which emerge from dying cells during the final stages of apoptosis; and exosomes (30–150 nm, though size can vary across studies), which are released from multi-vesicular bodies (18).

### 2.2 Exosomes in photodynamic therapy

Photodynamic therapy (PDT) employs photosensitizers (PSs) that, when activated by a specific wavelength of light in the presence of oxygen (O_2_), produce cytotoxic free radicals and ROS to destroy cancer cells (19, 20). However, free PSs in PDT often face limitations such as poor water solubility, photostability, aggregation, and off-target effects, which restrict their clinical applications (21, 22). Exosomes offer a promising solution by enhancing PS delivery to tumour cells, improving accumulation, and reducing systemic toxicity (23). Additionally, novel PDT strategies with PS agents can be developed via aggregation-induced emission (AIE) properties through ROS generation and tumour-targeted phototherapy (24, 25). Studies have demonstrated that the acidic tumour microenvironment, combined with laser irradiation and exosome-based carriers such as PMA/Au BSA@Ce6 or ChiP-Exo, can significantly enhance PDT efficacy via dual-stage light-directed subcellular destruction (26). The Exo-PMA/Au-BSA@Ce6 system is an advanced, exosome-based nanoplatform that elegantly combines enhanced tumor-targeted delivery, real-time fluorescence imaging, and potent photodynamic therapy. PMA/Au-BSA@Ce6 nanoparticles accommodate an amphiphilic polymer (PMA), ultra-small gold nanoparticles (Au), bovine serum albumin (BSA), and the photosensitizer chlorin e6 (Ce6) which were loaded into urinary exosomes via an instant electroporation technique, creating the hybrid Exo-PMA/Au-BSA@Ce6 nanovehicles. This nanoparticle structures got collapsed and released inside cancer cells under 633nm laser irradiation and acidic condition, producing considerable singlet oxygen, effectively inhibiting growth of tumor cells (27). Although photosensitizer-induced reactive oxygen species (ROS) are cytotoxic, their short lifespan and limited diffusion restrict the overall anti-tumor efficacy. To overcome this limitation, Zhao et al. (2021) developed a nucleus-targeted exosome engineered with a chimeric peptide (ChiP-Exo) to enhance photodynamic therapy (PDT). Using a dual-stage light strategy, they achieved sequential plasma membrane and nuclear degradation in cancer cells. This approach enabled *in situ* ROS activation at the nuclear level, leading to effective nuclei disruption, inhibition of tumor growth, and reduced systemic toxicity (28). ChiP-Exo’s plasma membrane-targeted PDT, for example, can degrade membrane structures, induce photochemical internalization (PCI), and promote lysosomal escape (26). Natural PSs like hypericin from Hypericum perforatum have also been investigated; to address their poor solubility, high lipophilicity, instability, and production cost, *H. perforatum*-derived exosome-like nanovesicles (HPDENs) have been introduced as an innovative PS platform for PDT (26). Furthermore, encapsulation within exosomes protects PSs from degradation, improving photostability and therapeutic efficiency (23). While exogenous nanocarriers such as hyaluronic acid, polydopamine, and chitosan have been explored to encapsulate PSs, they often face immune system clearance (26), whereas engineered exosomes loaded with PSs demonstrate low immunogenicity, high biocompatibility, and enhanced blood circulation, thereby improving PDT performance (23). To overcome challenges such as poor tumour targeting and limited tissue penetration of light, orchestrated nanoplatforms of indocyanine green (ICG) have been developed to improve bioavailability and tumour specificity in PDT (29). For instance, a novel bio-nanoplatform was developed by integrating edible ginger-derived exosome-like nanoparticles (GDNPs) with the photosensitizer indocyanine green (ICG), forming GDNPs@ICG. These nanoparticles were internalized by tumor cells through a lipid-dependent pathway. Upon 808 nm near-infrared (NIR) laser irradiation, GDNPs@ICG generated high levels of ROS, malondialdehyde (MDA), and local hyperthermia within the tumor, leading to lipid peroxidation and endoplasmic reticulum (ER) stress, thereby enhancing the efficacy of photo-mediated breast tumor therapy. Expression analyses of biomarkers such as CD31, N-cadherin, IL-6, IFN-γ, CD8, p16, p21, and p53 further demonstrated that GDNPs@ICG effectively reduced angiogenesis, suppressed metastasis, activated anti-tumor immune responses, and promoted tumor cell senescence (30). In another study, melanoma-derived exosomes were employed to design perfluorocarbon (PFC)-based drug nanocarriers co-loaded with ICG and camptothecin (CPT) (ICFESs), enabling targeted photochemotherapy (31). Similarly, a combinational therapeutic strategy was reported using tumor exosome-based nanoparticles co-formulated with ICG and the tyrosine kinase inhibitor gefitinib (IG@EXOs). This approach demonstrated enhanced antitumor efficacy against oral squamous cell carcinoma (OSCC) through synergistic phototherapy and molecularly targeted therapy (32).

Additionally, the synthesis of organic PSs capable of generating ROS from intrinsic non-photosensitizer fluorophores upon light irradiation is an emerging approach for effective cancer treatment (33). Addressing melanin interference in PDT, coordination-driven assembly of Ir (III) complex PSs with Fe (III) ions into nanopolymers, camouflaged with exosomes, has been shown to eradicate melanoma tumours and inhibit metastasis formation in mouse models (34).

### 2.3 Exosomes in immunotherapy

**Figure 2:**
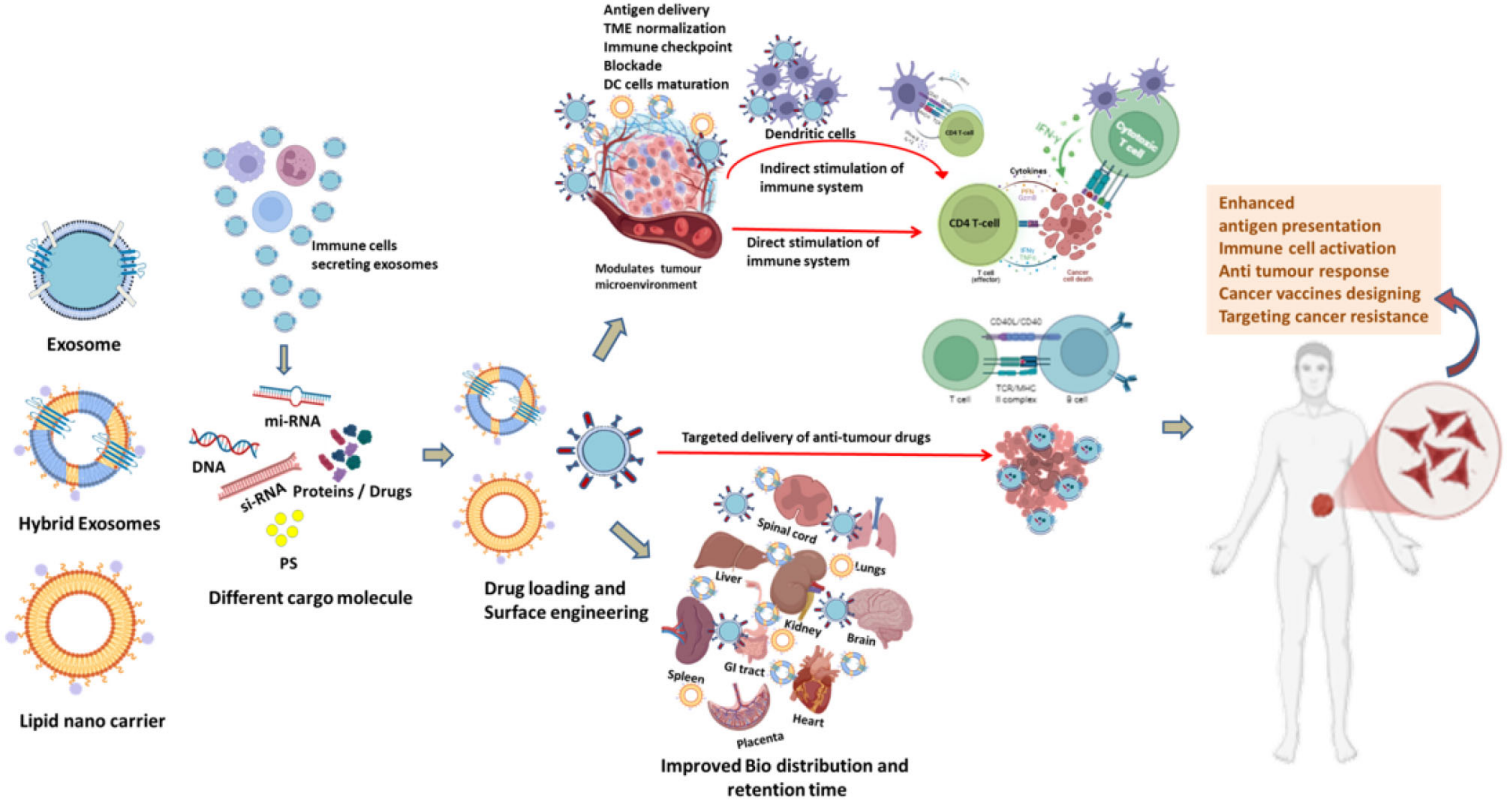
Schematic representation of improvement in Bio-distribution of exosomes in different human body organs and immune cell activation of exosomes and Lipid nano carriers directly, indirectly or targeted type to elicit specific immune response modulating TME to target cancer cells in cancer immunotherapy. DC (Dendritic cell), (TME) Tumour micro-environment.

Immunotherapy leverages the immune system to selectively eradicate cancer cells and offers advantages over conventional treatments, which often damage healthy tissues and promote drug resistance (35, 36). Exosome-based immunotherapy is emerging as a promising alternative due to its ability to deliver tumour-associated antigens, immune checkpoint inhibitors (ICIs), and immunomodulatory molecules with high specificity and low immunogenicity (9) (**Figure 2**). Nasopharyngeal carcinoma (NPC), a malignancy prevalent in Southeast Asia, is often diagnosed late and exhibits high recurrence and metastatic rates, compounded by resistance to chemo-radiotherapy and limited responses to immune checkpoint inhibitors due to T cell exhaustion and an immunosuppressive tumor microenvironment (TME). Exosomes, bilayered vesicles of 30–150 nm, play crucial roles in cell–cell communication within the TME, and tumor-derived exosomes (TEX) in NPC have been linked to angiogenesis, metastasis, and therapeutic resistance, though their role in immune evasion remains underexplored; importantly, their detectability in body fluids highlights their potential as biomarkers for early diagnosis and prognostication (37). Beyond NPC, exosomes are increasingly investigated as therapeutic platforms, such as in genetically engineered tumor cell-derived exosomes co-delivering endogenous tumor antigens and immunostimulatory CpG DNA, which enhanced dendritic cell activation and elicited robust antitumor immunity in murine melanoma models (38). Similarly, their unique lipid–protein composition and natural role in genetic material transport position exosomes as promising low-toxicity, high-efficiency vectors for gene therapy, although further work is required to optimize targeting and cargo loading (39). Moreover, innovations such as dendritic cell-mimicking nanovaccines (HybridDC), engineered with tumor-associated exosomes, costimulatory molecules, and CCR7, have demonstrated superior antigen delivery, improved lymph node targeting, and synergy with immune checkpoint blockade in glioma models, underscoring the potential of exosome-based strategies to reshape the immune landscape and enhance personalized cancer immunotherapies (40). Engineered exosomes, such as GEMINI-Exos armed with anti-CD3, anti-EGFR, PD-1, and OX40L, have demonstrated significant inhibition of triple-negative breast cancer in mice (41), while surface modifications like PEGylation or CD47 overexpression enhance circulation and tumour targeting (26). SMART-Exos displaying bispecific antibodies (anti-CD3/anti-EGFR or anti-CD3/anti-HER2) enable simultaneous T cell activation and redirection toward tumour cells, and CD40L-expressing exosomes further boost dendritic cell maturation and cytokine secretion (42). By overcoming tumour immune escape mechanisms and enabling precise modulation of the tumour microenvironment, exosome-based strategies including antigen delivery, immune checkpoint blockade, and TME normalization hold transformative potential for next-generation cancer immunotherapy (**Figure 1**).

### 2.4 Engineering exosomes for PDT and immunotherapy

#### 2.4.1 Advantages of engineered exosomes & its potential for PDT & immunotherapy

**Figure 3:**
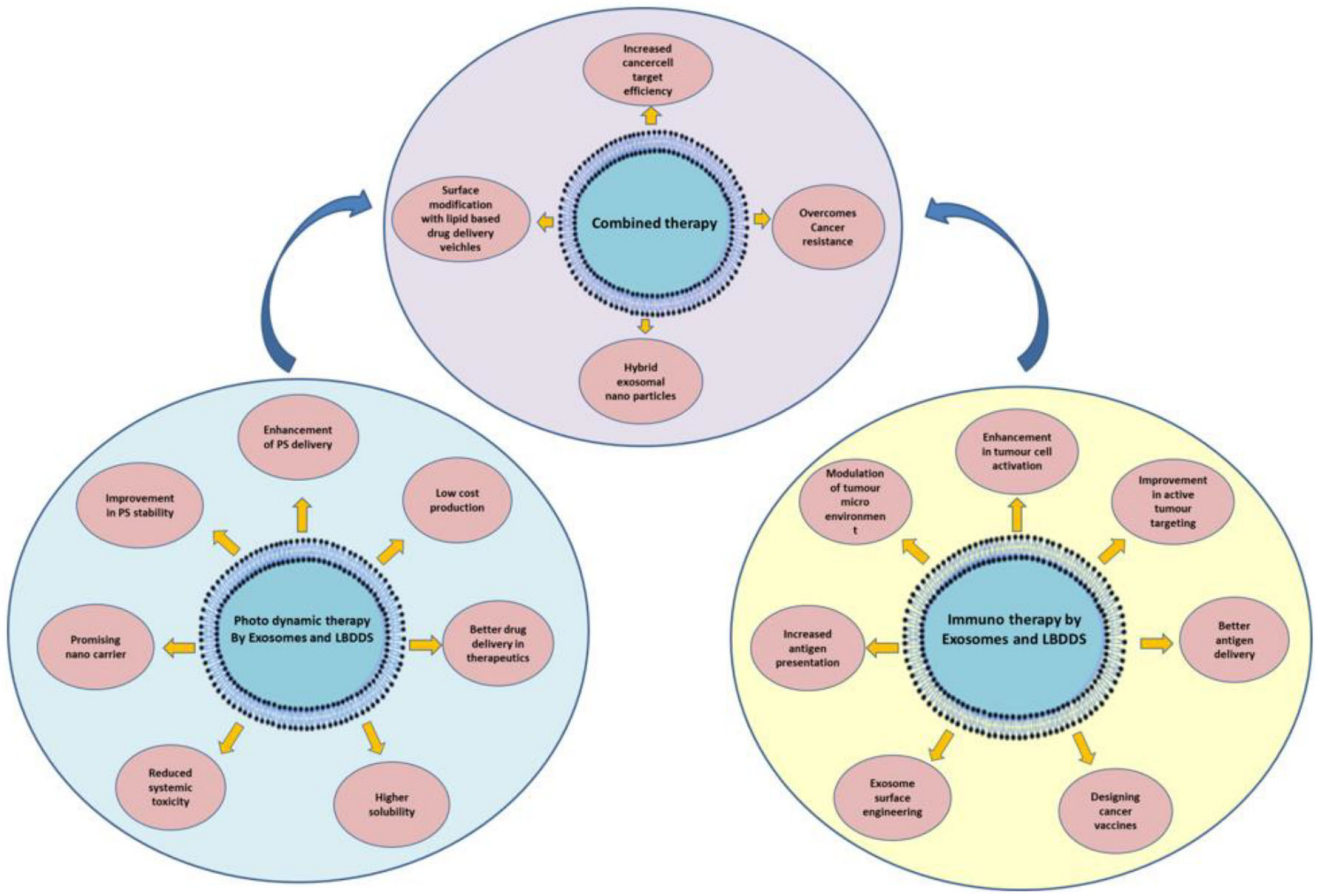
Combining the advantages of exosome and lipid-based nanocarriers in photodynamic therapy (PDT) and immunotherapy to enhance cancer cell targeting.

Engineered exosomes combine unique biological and physicochemical properties that make them highly attractive for targeted cancer therapy. Their nanoscale size (30–150 nm) facilitates deep penetration into tumor tissue via the enhanced permeability and retention (EPR) effect, while the native lipid bilayer provides structural stability and protects encapsulated cargo from enzymatic degradation during systemic circulation (43). Compared with synthetic nanocarriers, exosomes display low immunogenicity and high biocompatibility, thereby minimizing the risk of adverse immune reactions (44) (**Figure 3**) (**Table 1**).

**Table 1:** Comparision of therapeutic potential characteristics between exosomes and lipid nano-carriers.

Efficiency of drug carrier	Exosomes	Lipid nanocarriers
Drug-loading/Encapsulation Efficiency (EE)	Exhibit relatively low and variable drug-loading efficiencies. Using electroporation, ~20% doxorubicin encapsulation efficiencies were observed in reports (45). Bagheri et al. was able to depict ~35% EE with mesenchymal stem cell (MSC)-derived exosomes (46). In Optimized electroporation conditions, markedly improved vesicle recovery and doxorubicin loading efficiency was observed, with condition-dependent gains approaching ~50–60% in some cases (47). Nonetheless, vesicle source, cargo type, and loading strategies are highly influenced by encapsulation outcomes.	Consistent high EE due to well-developed active (remote) loading strategies. Doxorubicin EE showed ~98% using pH-gradient remote loading (48). Similarly, >90% EE was demonstrated with ammonium sulfate gradients (49).
Circulation Half-life (t½)	Intravenously administered native exosomes are rapidly cleared by the mononuclear phagocyte system; Only ~2–4 minutes of plasma half-lives have been reported in mouse studies (50) (51);. Very short circulation times and rapid hepatic and splenic uptake was also confirmed in a study report (52).	Prolonged systemic circulation may be obtained by PEGylated liposomes. For Doxil^®^ (pegylated liposomal doxorubicin), Gabizon et al. reported a terminal half-life of ~55 h in humans (53). FDA labeling indicates a range of 50–80 h, shows a longer order of magnitude than exosomes. This extended circulation largely prevented rapid clearance because of PEGylation.
Tumor Accumulation (%ID/g or relative uptake)	After IV administration unmodified exosomes show minimal tumor accumulation. Comparing exosomes and liposomes, exosomes show rapid clearance with little detectable tumor uptake, although intratumoral injection produced higher local retention than liposomes (54).	Enhanced permeability and retention (EPR) effect was exploited by PEGylated liposomes to achieve measurable tumor accumulation. Effective targeting of solid tumors by radiolabeled PEGylated liposomes in locally advanced cancer patients reported measurable tumor uptake in a clinical imaging study (55),. In a preclinical work, Tumor deposition of ^111In-labeled PEGylated liposomal vinorelbine, was quantified reaching 14.92 ± 3.96%ID/g in murine colon carcinoma models. Unmodified exosomes after intravenous delivery, usually seen with tumor uptake levels with a more rapid clearance.

A key advantage lies in their intrinsic homing capability, dictated by protein and lipid signatures inherited from donor cells. This property enables selective accumulation in tumors and other specific tissues without extensive chemical modification (9). Precision targeting can be further enhanced through surface engineering strategies such as ligand conjugation, genetic fusion of targeting motifs, or chemical modification, which improve tissue specificity, help bypass biological barriers, and minimize off-target effects (56).

Engineered exosomes also support multimodal therapeutic applications. Their membranes can simultaneously incorporate imaging agents and therapeutic cargo, enabling real-time biodistribution tracking alongside treatment delivery. This multifunctionality supports theranostic approaches, where diagnosis and therapy are combined within a single platform (14). Moreover, exosomes can accommodate a wide spectrum of hydrophilic, hydrophobic, and macromolecular therapeutics including nucleic acids, proteins, and chemotherapeutics providing versatility across oncology, immunotherapy, and regenerative medicine (**Figure 1**; **Figure 2**).

Over the past decade, significant progress has been made in designing exosomes for targeted cancer therapy with diverse payloads such as siRNA (57), miRNA (58), and chemotherapeutic agents (59). Their stability protects cargo from enzymatic degradation, while their innate homing and biocompatibility ensure safety and tolerability *in vivo* (43, 44). For instance, exosomes from breast cancer cells engineered to overexpress miR-134 suppressed Hsp90, inhibited invasion and migration, and enhanced sensitivity to anti-Hsp90 drugs. Similarly, endothelial cell-derived exosomes enriched with miR-503 reduced tumor cell proliferation and invasion *in vitro* (56). THP-1 macrophage–derived exosomes transfected with miR-143, when intravenously administered to colon cancer-bearing mice, elevated miR-143 expression in tumors, kidneys, and serum, resulting in significant tumor growth inhibition (43, 60–62).

Beyond miRNA delivery, genetically engineered exosomes have also been explored as immunotherapeutic platforms. Streptavidin-lactadherin (SAV-LA) expressing exosomes from B16BL6 melanoma cells, functionalized with biotinylated CpG DNA (CpG-SAV-exo), effectively activated dendritic cells, enhanced antigen presentation, and produced stronger antitumor responses than exosomes or CpG DNA alone (38). Similarly, PD1-engineered exosomes co-loaded with the immune adjuvant imiquimod (PD1-Imi Exo) demonstrated potential in augmenting checkpoint blockade therapy (63). Artificial exosomes derived from Siglec-10 engineered M1 macrophages, formulated into a hydrogel encapsulating the efferocytosis inhibitor MRX-2843, reprogrammed macrophage polarization and efferocytosis when combined with X-ray radiation, thereby enhancing phagocytosis, antigen presentation, and robust antitumor immunity in ovarian cancer (64).

The method of drug loading is another critical determinant of therapeutic efficacy. Cargo hydrophilicity, lipophilicity, molecular weight, membrane integrity, and vesicle stability collectively influence loading efficiency and release kinetics (65). For example, studies on milk-derived exosomes show that hydrophilic drugs achieve significantly higher loading rates (33–65%) compared to hydrophobic drugs (13–22%) [Milk-derived exosomes as a promising vehicle for oral delivery of hydrophilic biomacromolecule drugs]. Doxorubicin encapsulation efficiency varies by species and method, with goat-derived exosomes exhibiting favorable biphasic release profiles (66). Similarly, engineered exosomes demonstrated superior loading of hydrophilic porphyrins via saponin-assisted incubation and hypotonic dialysis achieving up to 11-fold higher efficiency whereas hydrophobic porphyrins consistently showed poor incorporation (67) (**Table 1**).

Engineered exosomes also serve as potent vehicles for apoptosis-inducing agents. TRAIL-loaded exosomes from mesenchymal stem cells (MSCs) have demonstrated strong cytotoxic activity against lung, pleural mesothelioma, renal, breast, and neuroblastoma cell lines (68, 69). Likewise, HEK293T-derived exosomes engineered to express Lamp2B fused with the IL-3 receptor, overexpressed in chronic myeloid leukemia (CML), have been loaded with imatinib or BCR-ABL siRNA. These IL3-Lamp2B (IL3L) exosomes showed enhanced tumor targeting and therapeutic efficacy in preclinical models (62, 69).

In immunotherapy, engineered exosomes are emerging as modular vaccine platforms. One strategy fused the ovalbumin antigen to the lipid-binding C1C2 domain of lactadherin, displaying the antigen on the exosome surface. When used as a DNA vaccine, this design elicited robust antigen-specific CD4^+^ and CD8^+^ T-cell responses, effectively suppressing tumor progression in fibrosarcoma, melanoma, and thymoma models. Drug-loaded exosomes modified with targeting ligands have also shown selective accumulation in tumors following intravenous administration, where doxorubicin- and imatinib-loaded constructs inhibited tumor growth without systemic toxicity (45, 69, 70).

Genetic engineering further expands the utility of exosomes as gene delivery systems. Ohno et al. demonstrated efficient delivery of let-7a miRNA to malignant cells by fusing platelet-derived growth factor with the GE11 peptide, thereby enhancing binding to EGFR-positive tumors and correcting miRNA dysregulation (56, 71).

Taken together, engineered exosomes offer a highly versatile platform for precision drug delivery, PDT, and immunotherapy. Their biocompatibility, stability, and ability to integrate diagnostic and therapeutic functions position them at the forefront of next-generation cancer therapeutics (**Table 1**).

#### 2.4.2 Key preclinical and clinical studies

A growing body of preclinical data underscores the translational promise of engineered exosomes in oncology. TRAIL-loaded MSC-derived exosomes have demonstrated potent induction of apoptosis in lung, breast, renal, neuroblastoma, and mesothelioma cell lines, with significant tumour regression observed in xenograft models (68). In hematologic malignancies, IL3-Lamp2B–engineered exosomes loaded with imatinib or BCR-ABL siRNA selectively targeted chronic myeloid leukemia cells, reducing tumour burden without overt systemic toxicity (69).

In solid tumours, GE11 peptide modified exosomes successfully delivered let-7a miRNA to epidermal growth factor receptor positive breast cancer cells, restoring tumour-suppressor miRNA levels and reducing proliferation *in vitro* and *in vivo* (71). Similarly, exosomes loaded with doxorubicin and modified with tumour-homing peptides showed preferential tumour accumulation, significant growth inhibition, and minimal cardiotoxicity compared to free drug administration (70).

A clinical pilot trial report explained plasma-derived exosomes from head and neck cancer patients undergoing PDT reflecting dynamic EMT-to-epithelial transitions, positioning them as potential biomarkers of therapeutic response (72). ROS-sensitive PEGylated exosomes for chemo-PDT could be applied as a preclinical advancement to engineered exosomes (73). Other studies include oral milk exosomes for brain-targeted PDT (74), and tumor-derived exosome hybrids co-loaded with photosensitizers and drugs for synergistic PDT chemotherapy (31). Together, these findings underscore exosomes as both biomarkers and multifunctional PDT carriers with high translational potential.

Early-stage clinical investigations are also underway. A first-in-human Phase I trial (NCT03608631) assessed Participants received mesenchymal stromal cells-derived exosomes with KrasG12D siRNA IV over 15–20 minutes on days 1, 4, and 10 with treatment repeatation in every 14 days for up to 3 courses in the absence of disease progression or unacceptable toxicity. Participants who responded were continued 3 additional courses (75). Another trial (NCT01159288) explored autologous dendritic cell–derived exosomes loaded with tumour antigens as a personalized cancer vaccine for non-small cell lung cancer, reporting induction of tumour-specific T-cell responses (76). An immunotherapy was developed by Gustave Roussy and Curie institutes involving metronomic cyclophosphamide (mCTX) followed by vaccinations with tumor antigen-loaded dendritic cell-derived exosomes (Dex). mCTX inhibits Treg (regulatory T-cells) functions restoring T and NK cell effector functions and Dex are able to activate the innate and adaptive immunity. The median progression-free survival (PFS) in patients responding or stabilized after 4 chemotherapy cycles ranges from 2 to 2.8 months. They proposed a maintenance immunotherapy in 47 advanced unresectable NSCLC patients responding or stabilized after induction chemotherapy with Dex-based treatment to improve PFS rate at 4 months in these patients (76).

Collectively, these studies illustrate the versatility, safety, and therapeutic potential of engineered exosomes, laying a robust foundation for their translation into precision oncology and next-generation immunotherapies.

## 3 Lipid-based nanocarriers

Lipid-based nanocarriers are nanoscale delivery platforms composed of biocompatible lipid components designed to encapsulate and transport therapeutic agents with enhanced pharmacokinetic and pharmacodynamic profiles such as stability, solubility, and targeted delivery profiles, while minimizing off-target effects (77, 78). Owing to their structural versatility, these systems can encapsulate both hydrophilic and hydrophobic molecules, enhance drug solubility, protect labile compounds from degradation, and enable targeted delivery, thereby reducing systemic toxicity (79, 80) They have been extensively utilized in cancer therapy, gene delivery, vaccine formulations, and other biomedical applications (13, 81). Lipid based drug delivery system is broadly classified into 3 types, namely, (i) emulsion type, (ii) vesicular system and (iii) lipid particulate system (82). Liposomes, solid lipid nanoparticles (SLNs) (11, 83), and nanostructured lipid carriers (NLCs), alongside a wider array of lipid-based drug delivery systems (LBDDS) such as lipospheres, lipid drug conjugate nanoparticles (LDCs), self-emulsifying formulations (SEFs), Pickering emulsions, dry emulsions, micro- and nano-emulsions, solidified reverse micellar solution (SRMS) tablets, herbosomes (84), Phytosomes (85) cryptosomes (86), niosomes (87, 88), ethosomes (89) bilosomes (90), and transferosomes (91) were some of the modulated form of the basic LBDDS catagory. These formulations employ diverse excipients such as triglyceride oils, mixed glycerides, lipophilic and hydrophilic surfactants, and water-soluble components, allowing high flexibility in drug formulation design (13, 81). Each of these nanocarriers offers unique advantages in drug loading, release kinetics, and stability (92, 93).

Liposomes are spherical vesicles composed of one or more phospholipid bilayers are widely used for delivering anticancer drugs, nucleic acids, and vaccine antigens (78, 94). SLNs, consisting of a solid lipid matrix stabilized by surfactants, provide high drug entrapment efficiency and controlled release (92). NLCs, the second generation of SLNs, incorporate a blend of solid and liquid lipids, improving payload capacity and preventing drug expulsion during storage (93, 95). Advances in lipid composition engineering, PEGylation, ligand-mediated targeting, and stimuli-responsive designs have further improved their therapeutic precision and clinical applicability (96, 97).

Lipid-based nanocarriers provide multiple advantages, including biocompatibility, ease of chemical modification, high physical stability, and the capacity to carry both hydrophilic and hydrophobic drugs (81, 98). They address key pharmaceutical challenges such as the poor solubility and limited bioavailability of hydrophobic drugs, while enabling fine-tuning for disease indication, administration route, stability, and therapeutic efficacy. Clinically, lipid-based formulations are widely deployed for topical, oral, pulmonary, and parenteral delivery with minimal systemic toxicity, in part by altering drug biodistribution to avoid sensitive organs. Liposomes, for instance, have been adapted to carry anti-tumour and antimicrobial agents, chelating agents, peptide hormones, enzymes, proteins, vaccines, and genetic material (98). Notably, lipid nanoparticles represent the first nanomedicine delivery system to achieve widespread clinical translation, successfully delivering anti-cancer, anti-fungal, and antibiotic drugs, as well as gene therapies and anti-inflammatory agents (78).Specifically PDT and immunotherapy based therapeutic approaches maximally employs Liposomes, Micelles, SLNs and LNPs for successful applications.

Specialized systems such as cochleates formed via precipitation of negatively charged lipids with cations have shown promise for targeted delivery applications (99). Despite these advances, key developmental challenges remain, particularly related to the diversity of encapsulated cargo and the lack of standardized characterization methods, which complicate stability assessment, classification, and regulatory approval pathways (91).

### 3.1 Formulation techniques and functionalization of lipid-based drug delivery systems

Formulation techniques and functionalization strategies are central to optimizing the therapeutic performance of LBDDS, aiming to maximize stability, enhance bioavailability, enable controlled release, and achieve targeted delivery while minimizing adverse effects (91). Functionalization focuses on surface modification to improve biodistribution, facilitate tissue-specific targeting, and enhance biological interactions.

#### 3.1.1 Formulation techniques

The formulation types of LBDDS are also categorized into 4 types (i,e, Type I, Type II, Type III and Type IV). Type I formulation consists of oils without surfactants (e.g., tri-, di-, and monoglycerides), Type II bears oils and water insoluble surfactants, Type III contains oils, surfactants, and cosolvents (both water-insoluble and water-soluble excipients) and Type IV can be prepared with water-soluble surfactants and co-solvents (91). The process of formation of these LBDDS are explained briefly here as this is beyond the scope of this manuscript.

Liposome Formation: Liposomes self-assemble from phospholipids in aqueous environments to form bilayer vesicles capable of encapsulating active pharmaceutical ingredients. Common preparation methods include film hydration, solvent evaporation, and reverse-phase evaporation (77).

Solid Lipid Nanoparticles (SLNs): Produced by emulsifying a solid lipid in a liquid lipid or aqueous phase under high shear, SLNs offer stable encapsulation for hydrophilic and hydrophobic drugs (92).

Micelle Formation: Amphiphilic surfactants or block copolymers self-assemble into micelles in aqueous media, enabling solubilization of poorly water-soluble drugs and improving their pharmacokinetic profiles (100).

Nanoemulsions and Microemulsions: Generated by emulsifying oils and surfactants in water under high shear, these systems produce stable nanoscale droplets that enhance solubility and stability of hydrophobic drugs (101).

Nanostructured Lipid Carriers (NLCs): Formulated by blending solid and liquid lipids, NLCs improve drug loading capacity and release profiles over SLNs, making them particularly suitable for lipophilic drugs (102).

#### 3.1.2 Functionalization strategies

Functionalization of lipid based nanocarriers are mainly achieved in the following ways.

Surface Coating: Functionalizing nanocarriers with biocompatible polymers such as polyethylene glycol (PEG) extends circulation time by reducing recognition and clearance by the mononuclear phagocyte system (103).

Targeted Functionalization: Conjugation of specific ligands or antibodies (e.g., folate, transferrin) to nanocarrier surfaces enables receptor-mediated uptake in tumour cells or inflamed tissues, improving specificity (104).

pH-Responsive and Enzyme-Responsive Functionalization: Engineering nanocarriers to release their payload in acidic tumour microenvironments or in the presence of specific enzymes allows spatially controlled drug release (105).

Dual or Multi-Functionalization: Combining multiple targeting moieties or therapeutic agents on a single nanocarrier platform enables combination therapy or multi-modal drug delivery with enhanced efficacy (106).

These formulation and functionalization strategies collectively enable the development of next-generation lipid-based delivery systems that offer improved therapeutic index, reduced systemic toxicity, and high precision in treating complex diseases, particularly cancer (107).

### 3.2 Applications of lipid-based nanocarriers in photodynamic therapy

The therapeutic efficacy of PDT is often constrained by poor solubility, rapid clearance, and low tumour selectivity of photosensitizers (108). Lipid-based nanocarriers have been employed to overcome these limitations by improving photosensitizer stability, enhancing tumour accumulation via the enhanced permeability and retention (EPR) effect, and enabling co-delivery of chemotherapeutics or immune modulators for synergistic effects (**Table 2**) (109, 110).

**Table 2:** Overview of traditional and combinational drug delivery systems in photodynamic therapy (PDT) and immunotherapy.

Traditional drug delivery system in PDT and immunotherapy			
Sl. no.	System type	Advantages	References
1.	Lipid-based (e.g., liposomes, nanoliposomes, exosomes), surfactant-based (niosomes), polymer-based (polymeric nanoparticles, micelles, dendrimers, nanogels), and inorganic (silver, gold, iron, ZnO, silica, quantum dots) nanosystem in PDT and immunotherapy	Enhancement in surface modification, permeability and retention effect for PS-loaded system in tumors cells better biodistribution of the encapsulated agents, decrease in nonspecific targeting and decrease or eliminate side effects	(1, 18, 23)
2.	Extra cellular vesicles	EV derived PS for efficient targeting to cancer cell	(2)
3.	Exosomes	Augmentation of Immunosuppressive tumour microenvironment, Immune checkpoint blockade and therapeutic cancer vaccines	(3)
4.	HER2-specific exosome (EXO)-T vaccine	Efficient against HER2-positive breast cancer	(5)
5.	Immune cell derived exosomes	Effective Anticancer therapy	(9)
6.	Cancer cell derived exosomes	Highly efficient targeted delivery, Protected packaging, reduction of side effects in cancer treatment	(10)
7.	Exosomes	Efficient targeting of chemotherapeutics	(14)
8.	Tumor‐exocytosed exosome	Efficient tumour penetration	(16)
9.	Hypericum Perforatum-Derived Exosomes	Effective Tumor Photodynamic Therapy	(19)
Combinational drug delivery system on PDT and immunotherapy			
1.	Photodynamic and immune-combination therapy. Tumour derived reassembled exosome [Chlorin e6-loaded R-Exo (Ce6-R-Exo)]	Better drug delivery carrier and immune-stimulation in Pancreatic cancer	(3)
2.	Photodynamic and immune-combination therapy ICG@MnO_2_@Exo-anti-PD-L1	Effective immunotherapy in non-small cell Lung cancer (NSCLC).	(4)
3.	Nanocomplex of D‐A coordinated Ir (III) complex with macrophage derived exosome in PDT	Successful Reprogramming of tumor‐associated macrophages and eradicating the tumors in mice	(20)
4.	Coordination-driven assembly of Ir (III) complex photosensitizers with Fe (III) ions into nanopolymers camouflaged exosomes for combined photodynamic therapy and chemodynamic therapy	Efficient Eradication of a melanoma tumour as well as inhibition of metastases	(21)
5.	Genetically engineered multifunctional exosomes	Effective Anti-cancer immunity	(28)
6.	Tumour derived exosomes	Effective Tumour immunotherapy	(27)
7.	CD47-expressing tumour-derived exosomes with cRGD-modified liposomes co-loaded with miR-497 and triptolide (TP) (miR497/TP-HENPs)	Enhanced tumour accumulation and induced apoptosis	(107)
8.	γδ-T cell–derived exosomes in combination with PDT	Effective Anti-tumour immunity	(108)

The table summarizes different nanosystems and extracellular vesicle (EV) based approaches, highlighting their specific advantages such as targeted delivery, improved biodistribution, enhanced therapeutic efficiency, and immune modulation.

#### 3.2.1 Liposomes

Liposomes have been extensively investigated for PDT applications, with formulations such as liposomal zinc phthalocyanine and verteporfin demonstrating improved pharmacokinetics and enhanced tumour phototoxicity (111, 112). A comprehensive review highlighted the role of liposomal formulations differing in size, composition, and surface modification (e.g., folate conjugation) in enhancing tumour targeting, reducing off-target toxicity, and improving PDT efficacy with photosensitizers such as chlorin e6, phthalocyanines, and porphyrins (113). Liposomal temoporfin (Foslip) demonstrated improved pharmacokinetics, enhanced tumour uptake, and reduced prolonged skin photosensitivity in preclinical and clinical evaluation, addressing a major limitation of conventional PDT (114). Similarly, a liposomal benzoporphyrin derivative monoacid ring A (BPD-MA, marketed as Visudyne) exhibited controlled biodistribution and an improved safety profile compared to free drug. Lipid-anchored BPD-liposome combinations achieved significantly greater PDT efficacy at lower light doses compared to either formulation alone.

#### 3.2.2 Solid lipid nanoparticles

SLNs represent another important lipid-based drug delivery system for PDT. For example, SLNs loaded with aluminum phthalocyanine chloride modulated immunogenic cell death in melanoma models (115). Hypericin (Hy), a natural phenanthroperylenequinone photosensitizer from *Hypericum perforatum*, shows therapeutic potential but suffers from hydrophobicity. Encapsulation into SLNs (<200 nm, ultrasonication-prepared) achieved high entrapment efficiency, enhanced photostability, and improved drug loading (116). Thermoresponsive solid lipid nanoparticles with non-covalently bound temoporfin (T-SLNP) exhibited faster accumulation kinetics and higher phototoxicity *in vitro*, and biodegradable nanosystems (<50 nm) based on polymer-surfactant stabilized T-SLNPs demonstrated improved *in vivo* anticancer efficacy compared with commercial temoporfin formulations, along with controlled release and superior biocompatibility (117). SLNs have also improved the solubility and PDT efficacy of photosensitizers such as SLN-AlPc, MPPa-loaded SLNs (115, 118) and verteporfin (119).

#### 3.2.3 Polymeric micelles

Micelles have also been widely explored for PDT. Thermosensitive mPEG-b-p(HPMAm-Lac2) micelles efficiently encapsulated hydrophobic Si(sol)2Pc photosensitizers, demonstrating high loading efficacy, controlled release, and strong photocytotoxicity (120). Polymeric micelles help address poor water solubility of many photosensitizers (121). For instance, DSPE-PEG2000 micelles trapped BODIPY3, yielding BODIPY3-PEG3 nanocomplexes with excellent solubility and stability in aqueous media (122). Micelles further extend circulation time by avoiding rapid recognition by proteins and macrophages (121). Encapsulation polymers include pluronics, PEG–lipid conjugates, and pH-sensitive systems such as poly(N-isopropylacrylamide) or polyion complex (PIC) micelles. Notably, imidazole-bearing ^1O2-responsive polymeric micelles allowed light-triggered on-demand delivery of photosensitizers, demonstrating stability during systemic circulation via ionic crosslinking (123).

#### 3.2.4 Nanostructured lipid carriers

NLCs have been used to increase drug-loading efficiency of hydrophobic photosensitizers such as curcumin and hypericin, thereby improving bioavailability and ROS generation (116, 124). A topical NLC formulation of 5-ALA for basal-cell carcinoma enhanced skin penetration and PDT effect (125). Targeted NLC approaches, such as Angiopep-2-modified Ce6-NLCs, demonstrated BBB penetration and enhanced PDT efficacy in glioblastoma (126). Natural lipid nanoparticles (LNPs) loaded with aluminum phthalocyanine showed significant therapeutic potential for melanoma PDT (127).

Targeted Lipid-Based Systems: Ligand-targeted liposomal PDT agents, including folate-conjugated formulations, selectively accumulated in cancer cells overexpressing folate receptors, thereby enhancing therapeutic specificity (128). This strategy exemplifies how lipid carriers can be engineered for precision targeting in PDT.

### 3.3 Applications of lipid-based nanocarriers in immunotherapy

Cancer immunotherapy seeks to harness the host immune system to recognize and eradicate malignant cells, using strategies such as immune checkpoint blockade, cancer vaccines, and adoptive T cell transfer (129, 130). Lipid-based nanocarriers have emerged as promising delivery platforms for immunotherapeutic agents, as they can encapsulate antigens, adjuvants, and immunomodulatory drugs, facilitate co-delivery to antigen-presenting cells (APCs), and modulate immune responses through controlled release and targeting (131, 132) (**Figure 2**).

#### 3.3.1 Liposomes

Liposomes have been widely explored in immunotherapy. They can deliver tumor-associated antigens (TAAs) along with Toll-like receptor (TLR) agonists to dendritic cells, eliciting robust antigen-specific cytotoxic T lymphocyte (CTL) responses (133). PEGylated and pH-sensitive liposomes enable efficient cytosolic delivery of nucleic acid vaccines (mRNA/DNA), thereby improving antigen expression and immunogenicity (133, 134). Gao et al. designed immune agonist-anchoring liposomes to co-deliver IL-2 and an anti-CD137 antibody, which promoted tumor infiltration of CD8+ T cells, enhanced cytokine and granzyme secretion, and elicited strong antitumor responses while reducing systemic toxicity (135). Another formulation, ILP (34A-PEG-ILP), conjugated to antibodies at the distal PEG end, demonstrated superior targeting efficiency to lung endothelial cells and tumour tissue compared with conventional liposomes (136). Liposomes are being developed to address challenges in cancer immunotherapy by enhancing vaccine efficacy through improved antigen delivery, normalizing the tumor microenvironment, modulating signaling pathways, and serving in combination regimens with chemotherapy, radiotherapy, and phototherapy (137). In addition, highly pH-sensitive polymer-modified liposomes prepared by surface modification of phospholipid vesicles with 3-methylglutarylated poly(glycidol)—facilitated endosomal escape and cytosolic delivery of antigenic molecules, proving effective in inducing antigen-specific immune responses (138). Archaeosomes (liposomes derived from archaeal lipids) present another innovative approach, capable of activating dendritic cells and enhancing adjuvant responses. Furthermore, liposomes have been integrated into multimodal strategies, combining photodynamic therapy (PDT) and photothermal therapy (PTT), to potentiate antitumor immunity (135).

#### 3.3.2 Micelles

Polymeric micelles provide another versatile lipid-based nanocarrier system in immunotherapy. PEG-polyglutamate micelles encapsulating IL-2 showed prolonged circulation and enhanced dendritic cell (DC) vaccine efficacy in tumor-bearing mice, leading to strong CTL responses. Similarly, micelles co-loaded with doxorubicin (DOX) and IL-12 plasmid DNA significantly outperformed single-agent formulations in inhibiting tumor growth. Micelles have also been engineered to deliver macrophage colony-stimulating factor (M-CSF), inducing T cell–mediated antitumor immunity, while SART3 peptide-loaded micelles promoted CTL and NK cell activity, along with enhanced DC infiltration into tumors. PEG-PLL-PLLeu micelles co-delivering STAT3 siRNA and ovalbumin upregulated DC activation markers (CD86, CD40) and IL-12 production, further boosting immune responses. Indoximod-based micelles co-loaded with DOX improved therapeutic efficacy by simultaneously inhibiting immunosuppressive pathways and augmenting chemotherapy. SLNs and NLCs have also been employed for the delivery of immune checkpoint inhibitors such as anti-PD-1 peptides and siRNAs, improving their stability and tumour accumulation (139) Other strategies have employed PEG-PE micelles as adjuvant carriers (e.g., MPLA for TLR signaling), or combination micelles (e.g., tranilast-, epirubicin-, or Doxil-based micelles) to enhance T cell infiltration and establish durable immunological memory in resistant cancers (140, 141). Advanced micelle platforms include IDO-responsive tryptophan-polymer micelles that disassemble in tumor cells to release IDO inhibitors, thereby recruiting effector T cells (142). Another self-assembled micelle system combined immunomodulators (epigallocatechin gallate palmitate and metformin) with DOX and immune checkpoint inhibitors to reduce PD-L1 expression and reshape the tumor microenvironment (143). Mannose-modified micelles have been optimized for DC targeting and vaccine delivery, with mixed micelles co-delivering ovalbumin and TLR-7 agonists showing robust antigen-specific humoral and cellular immunity (144). Additionally, inorganic nanovaccine micelles incorporating zinc-doped iron oxide nanoparticles successfully co-delivered peptide antigens and TLR3 agonists, stimulating potent immune responses (145).

#### 3.3.3 Solid lipid nanoparticles

SLNs have demonstrated significant promise in immunotherapy. SLN-AlPc formulations retained the activity of the hydrophobic photosensitizer aluminum phthalocyanine in aqueous media, inducing immunogenic cell death (ICD) and activating DCs in melanoma models (115). Cationic SLNs (cSLNs) have proven effective vaccine adjuvants, enhancing antigen uptake, BMDC activation, and memory immune responses in models of inactivated foot-and-mouth disease virus (146). Beyond vaccines, cSLNs have been used to encapsulate anticancer agents and proteins for improved *in vitro* and *in vivo* efficacy (81). P18 N PI ME-loaded SLNs demonstrated sustained release and improved PDT outcomes in cancer models (147), while SLNs also provided controlled release of immune suppressants such as MMF (148). Chitosan-coated AmB-SLNs enhanced macrophage cytokine responses (TNF-α, IL-12) (149), and actarit-loaded SLNs improved splenic targeting and retention *in vivo* (150). Collectively, these findings highlight SLNs as multifunctional carriers for peptides, proteins, small molecules, and vaccines (151). Solid lipid nanoparticles (SLNs) and nanostructured lipid carriers (NLCs) have also been employed for the delivery of immune checkpoint inhibitors such as anti-PD-1 peptides and siRNAs, enhancing their stability and tumor accumulation (139).

#### 3.3.4 Emerging concepts

Lipid-based micelles have also been adapted for anti-inflammatory roles, such as polymeric micelles carrying a Ru (CO)3Cl (amino acidate) segment for CO release, which attenuated LPS-induced monocyte inflammation (152). Recent studies highlight the integration of lipid-based nanocarriers with immune-stimulating PDT, termed photo-immunotherapy, where PDT-induced ICD is leveraged alongside nanocarrier-mediated delivery of immune adjuvants to amplify antitumor immune responses (153, 154). Such combined approaches represent a frontier in nanomedicine-driven immuno-oncology.

## 4 Combined applications of exosomes and LBDDS in PDT and immunotherapy

Exosomes and LBDDS possess bioactive cargos of proteins, nucleic acids, and lipids that naturally facilitate intercellular communication with intrinsic stability, low immunogenicity, biocompatibility, and efficient membrane penetration, making them attractive drug delivery systems (155). Many engineering strategies originally developed combined application for liposomes and exosomes such as sonication, extrusion, freeze thaw cycles, and microfluidic methods have been adapted for exosomes, improving their therapeutic potential.

### 4.1 Exosome–lipid hybrids in PDT and immunotherapy

Hybrid exosome–lipid formulations combine the biological advantages of exosomes with the tunable properties of lipid nanocarriers, improving drug loading, stability, targeting, and intracellular delivery. A notable example is the loading of indocyanine green (ICG) into hollow manganese dioxide (MnO_2_) nanospheres followed by encapsulation in PD-L1 monoclonal antibody–reprogrammed exosomes (ICG@MnO_2_@Exo-anti-PD-L1). This platform modulated the tumour microenvironment (TME) in non-small cell lung cancer by enabling synergistic PDT and immunotherapy: acidic pH triggered controlled anti-PD-L1 release, while MnO_2_ catalyzed H_2_O_2_-to-O_2_ conversion, alleviating hypoxia and enhancing T-cell activation (**Table 2**) (8). Photoimmunotherapy approaches have also leveraged γδ-T cell–derived exosomes in combination with PDT to potentiate antitumour immunity (156).

Exosomal lipid composition determined by parental cell type and physiological state—affects membrane curvature, cargo protection, and stability, making lipidomic profiling a potential diagnostic and therapeutic tool in oncology (155). Hybridization strategies such as fusing exosomal and endosomal membranes with pH-sensitive fusogenic peptides, introducing cationic lipids, or applying lipid extruders have enhanced cytosolic delivery of therapeutic cargos (157). For example, folate-modified lipid nano-assemblies (FD9R) combined with tumour-derived exosome inhibition and IRF3 silencing demonstrated synergy with immune checkpoint blockade in a murine breast cancer model (158). Similarly, incorporating the cationic lipid-sensitive endosomolytic peptide L17E into exosome-based systems promoted efficient cytosolic release of RNA therapeutics (159).

In hepatocellular carcinoma (HCC) models, a hybrid adipocyte-derived exosome platform co-assembled a ROS-cleavable docetaxel prodrug (DSTG) and a lipid-conjugated photosensitizer (PPLA) into lipid cores (HEMPs and NEMPs), which were encapsulated within exosome membranes. These hybrids exhibited significantly greater uptake efficiency in HCC cells compared with lipid-only nanoparticles (160).

### 4.2 Integration of exosomes with lipid-based systems

The integration of exosomes with lipid-based drug delivery systems (LBDDS) has enabled multifunctional platforms for PDT and immunotherapy. Methods such as fusion with fusogenic liposomes or assembly of lipid-enriched exosomal cargo using lipid extruders yield potent hybrid transport vehicles with synergistic therapeutic benefits (161, 162). Lipid nanoparticles can also be integrated onto exosome surfaces to improve targeting and delivery (162), while lipids themselves facilitate exosome biogenesis, secretion, and fusion with the multivesicular body (MVB) membrane. Lipid-rich exosomes, particularly those derived from the central nervous system, contain 1.5–3-fold higher ceramide (Cer), phosphatidylserine (PS), cholesterol, and sphingomyelin (SM) than other exosome types, reflecting parental cell origin and supporting cargo loading, endocytosis, macropinocytosis, and phagocytosis (163).

### 4.3 Bioinspired hybrid platforms

An important example of bioinspired design is the fusion of CD47-expressing tumour-derived exosomes with cRGD-modified liposomes co-loaded with miR-497 and triptolide (TP), producing hybrid nanoparticles (miR497/TP-HENPs) that markedly enhanced tumour accumulation and induced apoptosis (164).

Together, exosome lipid hybrids provide multifunctional delivery systems that unite natural biocompatibility with synthetic flexibility, enabling synergistic PDT and immunotherapy. Such approaches not only improve drug loading and targeting but also harness exosomal lipid biology to modulate tumour–immune interactions, marking them as promising candidates for next-generation cancer nanotherapeutics.

## 5 Therapeutic outlook and future directions

Hybrid exosomes combining lipid-based drug delivery systems (LBDDS) with native exosomes create drug carriers enriched with exogenous lipids while retaining the intrinsic biological properties of exosomes. Liposomes contribute chemical versatility, ease of large-scale production, extended shelf life, and circulation stability, while exosomes provide inherent biocompatibility, natural targeting ligands, and complex bioactive cargo (165). This synergistic integration holds promise for advancing clinical nanomedicine by delivering high drug payloads with precise tumour targeting, controlled release, stability under physiological stress, and minimal immunogenicity. The collective evidence strongly supports the potential of LBDDS exosome hybrids as transformative platforms for cancer therapy, including PDT and immunotherapy.

Although lipid-based nanocarriers have shown considerable success in PDT and immunotherapy, future development should focus on multifunctional hybrid platforms that integrate imaging, therapy, and immune modulation in a single nanosystem. In PDT, innovations such as NIR-responsive lipid carriers with enhanced photostability and tissue penetration, coupled with oxygen-generating or hypoxia-responsive elements, could overcome tumour microenvironment constraints (2, 166). In immunotherapy, the next generation of lipid nanocarriers may incorporate personalized tumour antigens and immune adjuvants with precision targeting ligands for dendritic cells or T cells, boosting antigen presentation and immune activation (133).

The integration of bioinformatics and AI-driven lipid formulation design could optimize nanocarrier composition and payload combinations for patient-specific therapies. Addressing challenges in scalable manufacturing, long-term stability, and regulatory harmonization will be critical for clinical adoption. Additionally, theranostic lipid-based nanocarriers capable of both therapy and real-time monitoring via incorporated imaging agents represent a promising direction for precision oncology.

## 6 Epilogue

The convergence of lipid-based nanocarrier technology and exosome-mediated delivery presents a compelling pathway toward next-generation targeted therapeutics. Lipid-based nanocarriers offer structural flexibility, high payload capacity, and facile surface modification, making them well-suited for applications in photodynamic therapy (PDT) and immunotherapy. Exosomes, in contrast, provide innate targeting, biocompatibility, and the ability to cross physiological barriers while evading immune clearance (167). Hybrid approaches such as synthetic lipid exosome chimeras or bioinspired lipid nanoparticles engineered to mimic exosomal properties promise to integrate the precision of synthetic nanocarriers with the natural communication networks of biological vesicles. These innovations hold potential to enhance biodistribution, therapeutic index, and patient outcomes.

Despite this promise, translating exosome-based PDT and immunotherapy into the clinic remains challenging. Preclinical data support their ability to simultaneously target tumors and sustain immune activation; however, regulatory uncertainties, the lack of standardized potency assays, and the complexity of reproducible large-scale manufacturing present significant barriers. Addressing these challenges will require proactive engagement with regulatory agencies, the development of scalable bioprocessing platforms, and rigorous quality control frameworks to ensure product consistency and safety. Furthermore, clinical trial designs that incorporate immune biomarkers and clearly demonstrate added value beyond conventional PDT or immunotherapy will be critical to establishing clinical relevance.

Ultimately, the successful clinical translation of hybrid exosome lipid systems will depend on aligning scientific innovation with pragmatic solutions to regulatory and manufacturing hurdles. By doing so, these platforms could advance from promising laboratory concepts to viable, multimodal cancer therapeutics that drive the next era of personalized medicine.
